# Vaspin inhibits kallikrein 7 by serpin mechanism

**DOI:** 10.1007/s00018-013-1258-8

**Published:** 2013-01-31

**Authors:** John T. Heiker, Nora Klöting, Peter Kovacs, E. Bartholomeus Kuettner, Norbert Sträter, Stephan Schultz, Matthias Kern, Michael Stumvoll, Matthias Blüher, Annette G. Beck-Sickinger

**Affiliations:** 1 Institute of Biochemistry, Faculty of Biosciences, Pharmacy and Psychology, Universität Leipzig, Brüderstraße 34, Leipzig, 04103 Germany; 2 Department of Internal Medicine, Universität Leipzig, Liebigstr. 20, 04103 Leipzig, Germany; 3 Center for Biotechnology and Biomedicine, Institute of Bioanalytical Chemistry, Faculty of Chemistry and Mineralogy, Universität Leipzig, Leipzig, 04103 Germany

**Keywords:** Vaspin, SerpinA12, Kallikrein 7, Crystal structure, Diabetes, Insulin

## Abstract

The molecular target of the adipokine vaspin (visceral adipose tissue-derived serpin; serpinA12) and its mode of action are unknown. Here, we provide the vaspin crystal structure and identify human kallikrein 7 (hK7) as a first protease target of vaspin inhibited by classical serpin mechanism with high specificity in vitro. We detect vaspin–hK7 complexes in human plasma and find co-expression of both proteins in murine pancreatic β-cells. We further demonstrate that hK7 cleaves human insulin in the A- and B-chain. Vaspin treatment of isolated pancreatic islets leads to increased insulin concentration in the media upon glucose stimulation without influencing insulin secretion. By application of vaspin and generated inactive mutants, we find the significantly improved glucose tolerance in C57BL/6NTac and *db/db* mice treated with recombinant vaspin fully dependent on the vaspin serpin activity and not related to vaspin-mediated changes in insulin sensitivity as determined by euglycemic-hyperinsulinemic clamp studies. Improved glucose metabolism could be mediated by increased insulin plasma concentrations 150 min after a glucose challenge in *db/db* mice, supporting the hypothesis that vaspin may inhibit insulin degradation by hK7 in the circulation. In conclusion, we demonstrate the inhibitory serpin nature and the first protease target of the adipose tissue-derived serpin vaspin, and our findings suggest hK7 inhibition by vaspin as an underlying physiological mechanism for its compensatory actions on obesity-induced insulin resistance.

**Electronic supplementary material:**

The online version of this article (doi:10.1007/s00018-013-1258-8) contains supplementary material, which is available to authorized users.

## Introduction

Obesity represents a fast-growing health problem that is reaching epidemic proportions worldwide [1] and is associated with an increased risk of premature death [2]. Obesity significantly increases the risk of developing type 2 diabetes mellitus, hypertension, coronary heart disease, stroke and several types of cancer [3]. Understanding the molecular mechanisms linking adipose tissue to these obesity related pathologies is of enormous scientific and medical importance. Cells of adipose tissue produce and secrete a variety of cytokines and cytokine-like molecules, termed adipokines, which have effects on insulin sensitivity, glucose and lipid levels, satiety and appetite [4].

Previously, visceral adipose tissue-derived serpin (referred to as vaspin in this paper; serpinA12 according to the serpin nomenclature [5]) was identified as a putative member of the serine protease inhibitor family, which was expressed in visceral adipose tissue of Otsuka Long-Evans Tokushima Fatty (OLETF) rats at the age when obesity and insulin plasma concentrations reach a peak [6]. Vaspin expression was shown to decrease with worsening of diabetes and body weight loss, whereas vaspin serum levels could be normalized by insulin or pioglitazone treatment. Consistent with that, we found increased vaspin mRNA expression in human adipose tissue associated with obesity, insulin resistance and type 2 diabetes [7].

Administration of vaspin to obese mice improves glucose tolerance, insulin sensitivity and altered gene expression of candidate genes for insulin resistance [6]. In addition, we could demonstrate that central vaspin administration leads to reduced food intake and has sustained blood glucose-lowering effects in mice [8], and this has since been confirmed in rats [9]. We and others have recently reported that elevated vaspin serum concentrations are associated with obesity and impaired insulin sensitivity in humans [10, 11].

It has therefore been postulated that increased vaspin expression and secretion could represent a compensatory mechanism associated with obesity, severe insulin resistance, and type 2 diabetes [6]. Althoughprotease inhibitor properties have been suggested as mechanism of vaspin action, until now a protease target of vaspin has not yet been identified. Here, we demonstrate the inhibitory serpin nature of vaspin by identifying human kallikrein 7 (hK7) as the first protease target of vaspin and confirm the vaspin–hK7 interaction in human plasma. We find human insulin as a substrate of hK7 and co-expression of vaspin and hK7 in pancreatic islets. We provide evidence that the serpin function of vaspin is essential for its physiologic effects and demonstrate that the anti-diabetic vaspin effects in vivo do not result from increased insulin sensitivity but are rather based on an insulin-stabilizing effect most likely by inhibiting hK7-mediated insulin degradation. Thus, our results give insight into the molecular mechanism of reported beneficial vaspin effects in obesity-related metabolic dysfunction.

## Materials and methods

### Generation of 10×His–vaspin fusion protein

Expression of native human vaspin, cloned into the pET-16b bacterial expression vector (10×histidin tag; Qiagen, Hilden, Germany), and variants vaspinT365R and vaspinA369P in *E. coli* BL21(DE3)pLysRARE was induced by the addition of isopropyl β-thiogalactopyranoside (1 mM) to the growth medium. Bacterial extracts were prepared using standard methods, and the fusion protein was purified by immobilized metal ion affinity chromatography (NiNTA FastStart kit; Qiagen) (Supplementary Fig. S1A–D). Protein identity and purity were affirmed by SDS–PAGE, HPLC, Western blot and MALDI-TOF mass spectrometry. ActiClean Etox affinity columns (Sterogene Bioseparations, Carlsbad, CA, USA) were used to remove potential endotoxin contaminations.

### Crystallization and X-ray structure determination

**Fig. 1:**
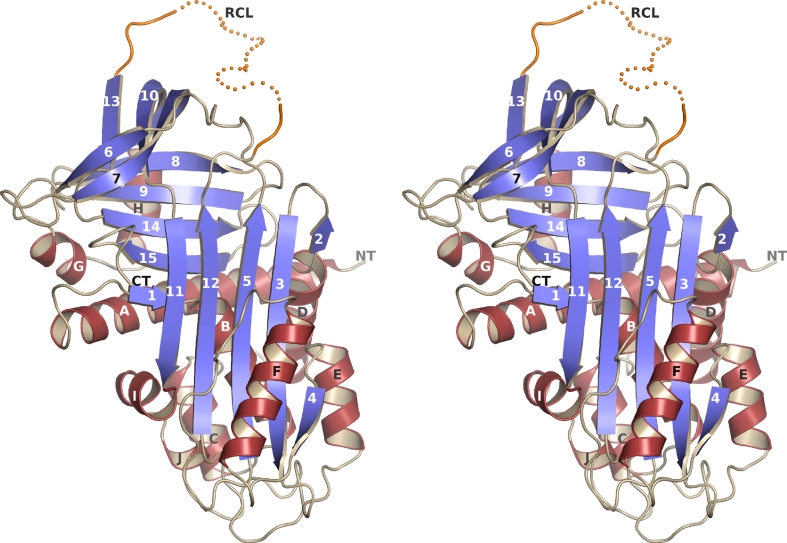
Crystal structure of vaspin. A stereo view of the better defined monomer A of the asymmetric unit. The residues of the reactive center loop (RCL) are illustrated as a *dotted line* and are flexible in the crystal structure (see also Supplementary Fig. S1F, G; Table 1)

Vaspin structure analysis was performed by crystallization and X-ray structure determination to 2.08 Ǻ data resolution. Prior to crystallization, the protein was subjected to a final gel filtration chromatography step (Superdex200 16/60 column; GE Healthcare, München, Germany; elution buffer containing 10 mM Tris pH 7.2 and 150 mM NaCl). Peak fractions were pooled and concentrated to about 8.4 mg/ml. In a hanging-drop vapor diffusion setup at 292 K, 1 μl protein and 1 μl reservoir solution were mixed and equilibrated against the reservoir solution of 0.5 ml containing 9 % (w/v) polyethylene glycol 4000, 0.1 M (NH_4_)_2_SO_4_ and 0.1 M sodium citrate pH 5.1. Crystals of up to 50 × 130 × 200 μm formed within 1–3 weeks (Supplementary Fig. S1E). The analyzed crystal was transferred stepwise to a buffer containing 20 % ethylene glycol in addition to the components in the crystallization buffer and frozen in liquid nitrogen. The diffraction data were integrated and scaled with the XDS [12] program package. The phase problem was solved by molecular replacement (program PHASER [13]) using the sequence-identity based truncated coordinates (program CHAINSAW [14]; side chains of non-conserved amino acids were truncated to the Cβ position) of human α_1_-antitrypsin (PDB id 1QLP) as the search model. The asymmetric unit contains two vaspin molecules. After modeling, refinement and validation (CCP4 programs [15] including COOT [16], REFMAC5 [17], BUSTER-TNT [18] and MolProbity [19]), the structure was deposited in the RCSB PDB as 4IF8. The final model comprises residues 37–366 and 378–414 of chain A and residues 35–277, 280–365 and 382–413 of chain B (full details in Table 1). Figure 1a was prepared using PyMOL (http://www.pymol.org).

**Table 1:** Details of data collection and refinement for vaspin crystal structure analyses

Space group	C2
Unit cell, *a* (Å)	133.6
*b* (Å)	152.1
*c* (Å)	61.5
*β* (°)	97.5
X-ray source	BESSY BL 14.2
Wavelength	0.91841
Temperature (K)	100
Resolution (Å)	29.8–2.08
Last shell (Å)	2.13–2.08
Completeness (%)	99.9 (100)^a^
Unique reflections	72,713
Multiplicity	4.3 (4.3)^a^
*R*_sym_ (%)	5.1 (48.3)^a^
*R*_rim_ (%)^c^	5.7 (54.6)^a^
Average *I*/*σ* (I)	19.5 (3.4)^a^
Wilson *B* (Å^2^)	40.2
Mean *B* (Å^2^)	52.9
Number of residues and mean *B* value (Å^2^) in brackets	
Chain A	367 (38.5)
Chain B	361 (68.4)
Sulfate	2 (66.3)
Water	224 (42.8)
*R*_work_/*R*_free_ (%)	20.3/22.2
r.m.s.d. bonds/angles (Å, °)	0.01/1.08
All-atom contacts clashscore	5.25
Molprobity score	1.69

^a^Values in parentheses refer to the highest resolution (last) shell

### Protease activity assays for hK4, hK5 and hK7

The activity assay for human recombinant hK4, hK5 and hK7 (all from R&D Systems, Minneapolis, MN, USA) were performed according to the manufacturers’ protocols using the recommended fluorogenic peptide substrates ES011 (hK4, hK5) and ES002 (hK7) (all from R&D) with minor modifications. Recombinant hK4 and hK7 were activated by thermolysin (R&D Systems) prior to the activity assay according to the manufacturers’ protocol. The stoichiometry for the reaction of recombinant vaspin with hK7 was determined as described previously [20]. Briefly, to a fixed concentration of protease hK7 (19 nM) increasing concentrations of vaspin were added to give molar ratios of inhibitor to protease of up to 5 in a final volume of 55 μl. After incubating for times sufficient to complete the reaction (>95 %), 25 μl of each reaction mixture was transferred to a 96-well plate and 75 μl of substrate solution (13.3 μM) were automatically injected into each well. The residual enzymatic activity was measured in duplicates from the initial linear rate of change of emission at 405 nm. The decrease in protease activity with increasing molar ratio of inhibitor/protease was fit by linear regression to obtain the stoichiometry of inhibition (SI) from the abscissa intercept. Association rate constant for the reaction of vaspin and hK7 were measured under pseudo-first-order conditions by using a tenfold molar excess of vaspin. Plots of remaining hK7 activity versus time demonstrated exponential loss of activity and the *k*_ass_ kinetic constant was determined based on the equations ln [*E*] = −*k*_obs_ · *t* and *k*_ass_ = *k*_obs_/[*I*] by linear fit to a plot of ln activity versus time [20].

### Analysis of vaspin–hK7 complex formation and in vitro digestion of native human insulin by hK7

For Western blot analysis, recombinant human vaspin and vaspin variants were incubated with or without hK7 at a molar ratio of 1:1 at RT. At indicated time point, SDS-samples were taken and ~200 ng total protein per lane were separated by SDS–PAGE and analyzed after Western blotting using primary antibodies for vaspin (Adipogen, South Korea), His-Tag (Abcam) or hK7 (Uscn Life Science, Wuhan, China). To isolate vaspin–enzyme complexes in human serum and plasma samples recombinant vaspin was added and allowed to form complexes and then isolated by affinity purification via its His-tag. Therefore, 25 μg of recombinant vaspin was added to 500 μl serum or plasma and incubated for 90 min at RT without protease inhibitors and with or without immediate addition of 100 μl Ni–NTA bead slurry (Abcam). After 90 min, 100 μl of Ni–NTA bead slurry and protease inhibitor cocktail were added to those samples without Ni–NTA beads and they were were incubated overnight at 4 °C on a rotator. Serum and plasma samples without vaspin, but otherwise treated identically, served as control for the affinity pull-down. After incubation times, the Ni–NTA beads were washed three times using a buffer with low concentration of imidazole (TBS, 150 mM imidazole, pH 8) and affinity purified protein complexes were eluted using Lämmli buffer containing 500 mM imidazole. Elution fractions were separated by SDS–PAGE and analyzed by Western blot using above mentioned antibodies.

For MS analysis, recombinant human vaspin was incubated with or without hK7 at a molar ratio of 5:1 for 24 h at RT to allow for vaspin–hK7 complex formation with respect to the observed complex stability in SDS–PAGE analysis. Reaction mixtures were desalted using ZipTip C18-filter tips (Millipore) and analyzed by MALDI-TOF MS and MSMS using LIFT mode on a Bruker Ultraflex III MALDI TOF/TOF mass spectrometer. Native insulin (Insuman Rapid; Sanofi-Aventis, Paris, France) was incubated at 0.75 mg/ml with activated 0.02 mg/ml hK7 for 60 min at 37 °C. HK7 activity was terminated by addition of 50 μM Pefabloc SC (Roche) and insulin fragments were reduced with 5 mM TCEP for 1 h at RT. Digests were analyzed by MALDI-TOF mass spectrometry and resulting peaks were further analyzed by MSMS mass spectrometry. Peak lists of MSMS spectra were searched against the SwissProt database using the Mascot search engine (Matrix Science, London, UK; http://www.matrixscience.com) in order to identify peptide fragments. For database searches, the following parameters were used—species: homo sapiens; digestion: none; monoisotopic masses; variable modification: methionine residues oxidized; mass tolerance MS: 100 ppm; mass tolerance MSMS: 0.5 Da.

### Animals

All animal studies have been performed in accordance to the *Guide for the Care and Use of Laboratory Animals* (NIH Publication No. 85-23, revised 1996) and were approved by the local authorities of the state of Saxony, Germany, as recommended by the responsible local animal ethics review board.

### Vaspin intraperitoneal (i.p.) treatment glucose tolerance test (GTT) and euglycemic-hyperinsulinemic clamp studies

Before i.p.- GTT and euglycemic-hyperinsulinemic clamp studies, mice were given two i.p. injections (2200 hours, and then at 0800 hours the following day before the procedure) of pyrogen-free saline or vaspin (1 mg/kg body weight). GTT was performed as described previously [21]. Euglycemic-hyperinsulinemic clamp studies were performed on female *db/db* mice (BKS.Cg-*m+/+Lepr* ^*db*^/BomTac; Taconic, Lille Skensved, Denmark) at the age of 16 weeks. After an overnight fast (12 h), catheter implantation and hyperinsulinemic-euglycemic clamps were performed as described previously [22]. In brief, the insulin clamp was conducted with a continuous infusion of human insulin at a rate of 60 mU/kg/min to lower plasma glucose levels within a physiological range (~5 mmol/l). Physiological blood glucose concentrations were maintained by adjusting infusion of a 20 % glucose solution. Steady state was ascertained when glucose measurements were constant for 20 min at a fixed glucose infusion rate and was achieved within 120–240 min. Steady state was maintained for 45 min and blood samples (10 μL) were taken at 0 and 5 min, and then at 10-min intervals after reaching steady state. All infusions were done using micro-dialysis pumps (TSE Systems, Chesterfield, MO, USA).

### Pancreas histology and immunofluorescence

Pancreatic islets were isolated from 12-week-old C57BL/6JRj mice (*n* = 3) which were bred and housed in the Animal Laboratories at the University of Leipzig, Germany, under strict hygienic conditions. Mice were maintained at a 12-h light/dark cycle (0500/1700 hours) and had free access to food (V1536; Ssniff; Soest, Germany) and water. The pancreas was fixed with 4 % buffered formaldehyde, embedded in paraffin and cut in 7-μm-thick serial sections, which were either stained with hematoxylin–eosin (H&E) or immunostained for vaspin (Adipogen, South Korea) or hK7 (Uscn Life Science). As secondary antibodies, HRP coupled anti-rat immunoglobulins (AbD Serotec, Oxford, UK) and Envision dual link system-HRP (Dako, Glostrup, Denmark) were used as described previously [23]. Fluorescence immunohistology was performed on frozen pancreas sections of C57BL/6JRj mice. Slides were air-dried and fixed in ice-cold acetone for 10 min. Five slides, from various levels of the tissue, were stained with H&E for the evaluation of islets. Primary antibodies against vaspin (Adipogen) and insulin (Santa Cruz Biotechnology, Heidelberg, Germany) were used. The fluorochrome conjugated secondary anti species antibodies, green (FITC-derived) and red (Cy3-derived), were from Zymed (Invitrogen, Karlsruhe, Germany). As negative controls, sections were incubated without primary antibodies to identify nonspecific binding (Supplementary Fig. S6B, C).

### Islet isolation, Western blot and in vitro insulin secretion analysis

Islets of Langerhans were isolated from pooled pancreases of 12-week-old male C57BL/6JRj mice (*n* = 15). Pancreatic islets were prepared by fractionated collagenase digestion (Serva, Heidelberg, Germany) after separation from exocrine tissue and hand-picked under a stereomicroscope as previously described [23, 24]. For Western blot analysis, 250 islets were lysed in 100 μl lysis buffer (20 mM Tris (pH 7.8), 150 mM NaCl, 0.5 % NP-40, protease inhibitor cocktail (Pierce) and 20 μg total protein were loaded per lane. For insulin secretion analysis, following islet isolation, groups of five islets were incubated with glucose concentrations of 5.5 mmol/l under sterile condition in RPMI 1640 medium supplemented with 10 % fetal calf serum, 20 mmol/l l-glutamine, 100 U/ml penicillin and 100 g/ml streptomycin (all from Sigma-Aldrich, Munich, Germany) for 24 h at 37 °C under a humidified gas atmosphere (5 % CO_2_:95 % O_2_). Islet function was tested for each single experiment by islet insulin content after incubation experiments using different glucose concentrations (5.5, 11.1, 22.2 mmol/l). For insulin release assays, 5 islets were statically incubated in Krebs–Ringer buffer and stimulated for 2.5 h at 37 °C with various glucose concentrations (5.5, 11.1, 22.2 mmol/l) with or without vaspin (1 μg/100 μl and 10 ng/ml medium). Supernatant was collected and assayed for insulin content by ELISA. Islets were then sonicated in acid–ethanol solution and solubilized over night at 4 °C before assaying total insulin content using Ultra Sensitive Mouse Insulin ELISA Kit (Crystal Chem, Downers Grove, IL, USA).

### Statistical analyses

Data are given as mean ± standard errors of the mean (SEM) unless stated otherwise. Datasets were analyzed for statistical significance using a two-tailed unpaired Student’s *t* test, or differences were assessed by one-way ANOVA corrected by Bonferroni–Holm using the Statistical Package for Social Science, v.14.0 (SPSS, Chicago, IL, USA). *p* values <0.05 were considered significant.

## Results

### Vaspin crystal structure

Based on sequence homology with α_1_-antitrypsin vaspin has been predicted to belong to the serpin protein family [6]. After expression, purification and crystallization of recombinant human vaspin (Supplementary Fig. S1A–E), X-ray structure determination confirmed the typical structural core domains of serpins consisting of three β-sheets and nine α-helices (Fig. 1). As expected, nearby regions match closely in a superposition onto related serpins (Supplementary Fig. S1F, G). The fold corresponds to that of free serpins or serpins involved in a transient complex, i.e. the long central β-sheet consists of five strands. The reactive center loop (RCL) (extending from G_364_ to P_381_) is flexible in the vaspin structure. Details of data collection and refinement are listed in Table 1.

### Vaspin inhibits hK7 via serpin mechanism

**Fig. 2:**
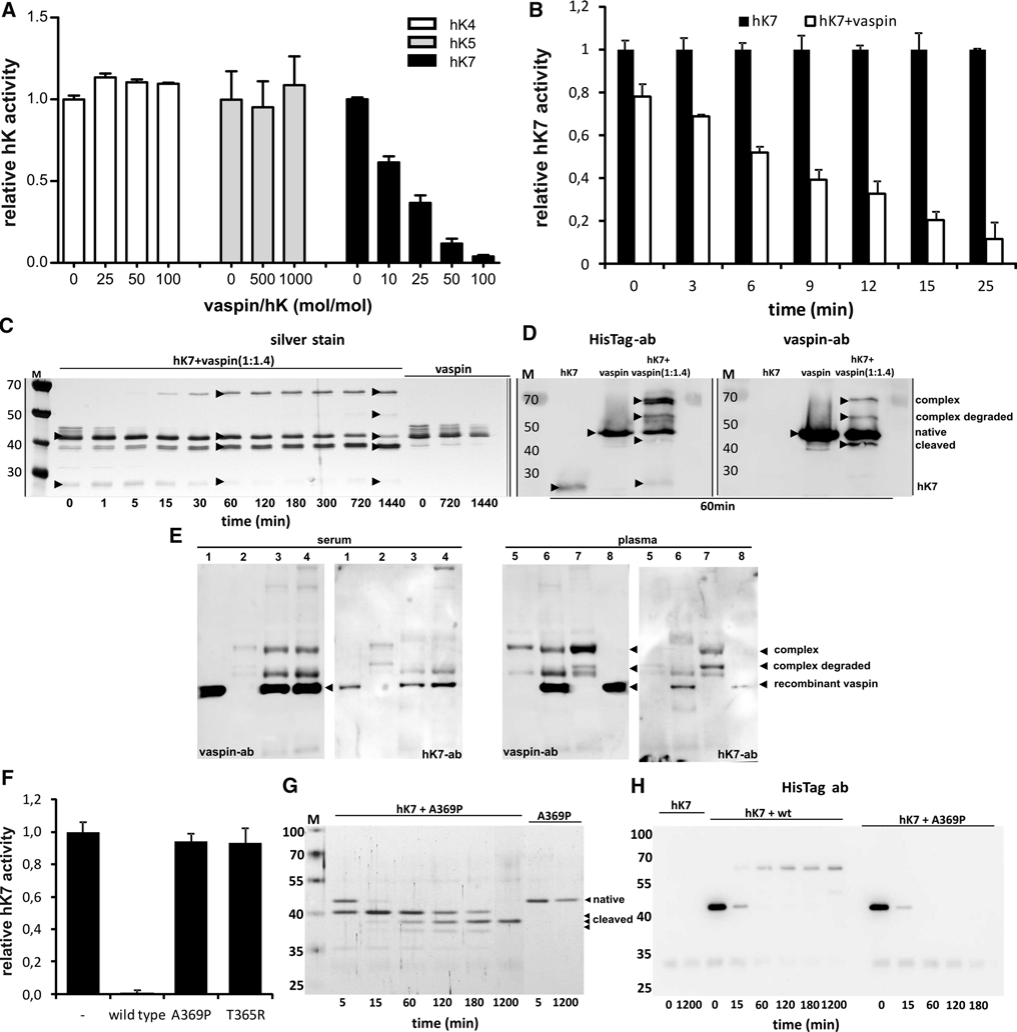
Identification of hK7 as protease target of vaspin. **a** Remaining proteolytic activity of closest related kallikrein members hK5, hK4 and hK7 after incubation with indicated molar excess of recombinant vaspin (10 min at RT). **b** Time course of hK7 inhibition with or without tenfold excess of vaspin (for stoichiometry and association rate constant of inhibition see Supplementary Fig. S2). **c** SDS–PAGE analysis and silver staining demonstrating vaspin–hK7 complex formation and stability over a period of 24 h (times presented in minutes). hK7 and vaspin (ratio of 1:1.4) or vaspin alone were incubated for the indicated time. **d** Immunodetection of vaspin, vaspin–hK7 complex and cleaved vaspin using anti-HisTag or anti-vaspin antibody. **e** Affinity purified vaspin–hK7 complex from human serum and plasma as detected by Western blot using anti-vaspin and anti-hK7 antibodies (*lane 1/8* rec. vaspin control, *lane 2/5* Ni–NTA bead control, *lane 3/6* vaspin and Ni–NTA beads simultaneously, *lane 4/7* vaspin and Ni–NTA beads successively). **f** Non-inhibitory variants vaspinT365R and vaspinA369P fail to inhibit hK7 proteolytic activity (tenfold excess, 60 min at RT). **g**, **h** Non-inhibitory vaspin variants are unable to form serpin–hK7 complexes and serve as substrates only, as demonstrated for vaspinA369P by SDS–PAGE and Western blot analysis. Results are expressed as the percentages of the value in the absence of vaspin (**a**, **b**, **f**), each *bar* representing the mean ± SD (*n* = 2–5) (**c**–**e**, **g**, **h**). Data shown are representative of at least two independent experiments

In an initial screening for a target protease, vaspin failed to inhibit serine proteases trypsin, elastase, urokinase, collagenase, factor Xa and dipeptidyl peptidase [6]. Instead of screening a broad range of proteases, we decided to focus on the kallikrein (hK) family, since vaspin has been shown to co-localize with kallikrein 5 (hK5) in human skin [25]. hK4, hK5 and hK7 are closely related evolutionarily [26] and were therefore tested in an initial screening. Primary investigation of hK4, hK5 and hK7 inhibition by vaspin was performed by monitoring protease activity over a range of molar ratios of serpin to protease using adequate fluorogenic peptide substrates. While vaspin failed to inhibit proteolytic activity of hK4 and hK5 (Fig. 2a), hK7 activity was significantly inhibited by vaspin in a time- and concentration-dependent manner (Fig. 2a, b). We determined the SI for the inhibition of hK7 by vaspin by incubating increasing ratios of vaspin to hK7 until completion of the reaction (residual hK7 activity <95 %) and obtained an SI of 3.4 ± 0.03 (Supplementary Fig. S2A). The association rate constant, *k*_ass_, for the reaction of vaspin with hK7 was determined under pseudo-first order conditions as 7.3 ± 1.8 × 10^3^ M^−1^ s^−1^ (Supplementary Fig. S2B).

Members of the serpin family typically form SDS-stable complexes with target proteases. To investigate complex formation and stability of the serpin–enzyme complex (SEC), we incubated hK7 and vaspin at a molar ratio of 1/1.4. Consistent with the observed hK7 inhibition progress by vaspin, the proteins formed stable complexes with an apparent molecular weight of ~70 kDa as detected by silver staining of SDS–PAGE gels (Fig. 2c), anti-vaspin and anti-HisTag Western blot (Fig. 2d) and MALDI-TOF mass spectrometry (Supplementary Fig. S4B). The complexes were stable under reducing conditions. Complex formation was detectable after 5 min, reached a plateau after 60 min and remained level for at least 24 h without significant complex dissociation (Fig. 2c). In parallel, the decrease in intensity of the intact vaspin band (~47 kDa) and increase of a ~42 kDa band mainly results from cleavage after a tyrosine residue within the artificial His-tag sequence of recombinant vaspin and only to minor extent from complex dissociation (Fig. 2c) as revealed by Western blot analysis (Fig. 2d). The slightly lower migrating band of cleaved vaspin due to slowly ongoing serpin–protease deacylation is only faintly detectable using the anti-His-tag antibody, while the tag-cleaved vaspin is detected as a clear band when using an anti-vaspin antibody (Fig. 2d). Furthermore, an additional band representing the degraded complex is detectable in the silver-stained gel and Western blot analysis (Fig. 2c, d), which is not unusual given an increased proteolytic susceptibility of the protease in complex with the serpin [27]. The control lanes of either vaspin or hK7 alone showed no additional bands to the native protein bands and neither hK4 nor hK5 were able to form complexes (unpublished observation).

We have tried to isolate endogenous vaspin–hK7 complexes from human serum and plasma, as well as lysates from isolated murine islets, but due to the low expression of both proteins in the samples and the potentially short half-life of the complex due to degradation or clearance, we were not able to detect endogenous vaspin–hK7 complexes by Western blotting. After addition of recombinant vaspin to human serum and plasma samples, complex formation by vaspin and very likely hK7 in human plasma could be detected as identified by Western blotting after affinity pull-down via the His-tag (Fig. 2e). The hK7 antibody primarily detects a band migrating below the full complex, most probably due to degradation of the exposed protein in the complex (protease inhibitors were not added to the reaction mixture, since protease activity is needed for complex formation) or better epitope accessibility for the antibody (Fig. 2e). We have observed a comparable complex degradation product for the in vitro formed complex of vaspin and hK7 as a band with a similar molecular weight in the Western blots shown in Fig. 2d. Interestingly, complex formation was only marginally observed in human serum, suggesting that circulating hK7 is lost during blood clotting.

X-ray structures of SECs demonstrate that after initial cleavage of the RCL remaining residues beginning at the P15 glycine, following the standard nomenclature [28], are inserted into the β-sheet A as a new β-strand [27, 29]. The P15 glycine is located in a tight turn between anti parallel β-strands and is highly conserved within the serpin family (Supplementary Fig. S1G) [30], most likely because torsion angles in this conformation are only allowable for the least restricted amino acid glycine in the Ramachandran plot. It has been shown that the serpin inhibitory mechanism is critically dependent on the length of the RCL [31] and is furthermore structurally based on conserved small side-chain amino acids within the hinge region of inhibitory serpins (Table 2). This also applies to vaspin, as the mutation of alanine 369 to proline (vaspinA369P) within the vaspin hinge region converts vaspin from inhibitor of hK7 to substrate (Fig. 2f–h). Analogous results have been shown for other serpins, such as antithrombin and antitrypsin [32, 33]. The variant vaspinA369P fails to inhibit hK7 activity (Fig. 2f) and the mutation prevents complex formation with the protease (Fig. 2f, g). Furthermore, inhibitory serpins feature a non-charged amino acid at position P14 in contrast to a charged amino acid at this position within non-inhibitory serpins [34, 35]. The charged residue at P14 has been demonstrated to significantly hinder RCL insertion into the central β-sheet [36]. The exchange of P14 threonine to arginine (vaspinT365R) resulted in a non-inhibitory vaspin variant (Fig. 2f), serving as hK7 substrate with no detectable complex formation (Supplementary Fig. S3).

**Table 2:** Reactive center loop (RCL) sequences for inhibitory human serpins and generated vaspin variants from residue P15 to P4′

		RCL	
		(**P15**-----------**P1**–	-**P1'**–P4')
SerpinA1	Antitrypsin	**G**TEAAGAMFLEAIP**M**–	–**S**IPP
SerpinA1_variant_	Pittsburgh	**G**TEAAGAMFLEAIP**R**–	–**S**IPP
SerpinA4	Kallistatin	**G**TEAAAATTFAIKF**F**–	–**S**AQT
SerpinA12	Vaspin	**G**TEGAAGTGAQTLP**M**–	–**E**TPL
SerpinC1	Antithrombin	**G**SEAAASTAVVIAG**R**–	–**S**LNP
SerpinE1	PAI–I	**G**TVASSSTAVIVSA**R**–	–**M**APE
SerpinA12 variants	VaspinA369P	**G**TEGA**P**GTGAQTLP**M**–	–**E**TPL
	VaspinT365R	**GR**EGAAGTGAQTLP**M**–	–**E**TPL

Comparison of the RCL sequences of vaspin and other prominent serpins suggested methionine 378 as P1 residue (Table 2; Supplementary Fig. S4A), though tyrosine is the preferred P1 residue [37]. Indeed, we could confirm methionine 378 as P1 residue for hK7 in vaspin by mass spectrometry (Supplementary Fig. S4B, C).

Thus, hK7 represents the first target protease of vaspin, demonstrating functional relevance of vaspin as an inhibitory serpin.

### hK7 cleaves human insulin within A and B chain

Interestingly, a study on the substrate specificity of recombinant hK7 used the oxidized form of the bovine insulin B chain as a model substrate, revealing main cleavage sites at tyrosine 16 and tyrosine 26 [38]. Here, we confirm these cleavage sites for human insulin under native conditions (Supplementary Fig. S5A). In vitro cleavage of insulin by hK7 was analyzed by MALDI-TOF mass spectrometry (Supplementary Fig. S5B) and peak identities of insulin fragments were confirmed by MALDI-TOF–MSMS. We could further identify a cleavage site at tyrosine 14 in the insulin A chain, which has been previously unknown (Supplementary Fig. S5A). A cleavage site after cysteine 7 in the B chain was only found under conditions of prior insulin reduction (unpublished observation). Accordingly, human insulin serves as a substrate of hK7.

### Vaspin and hK7 are expressed in pancreatic islets

**Fig. 3:**
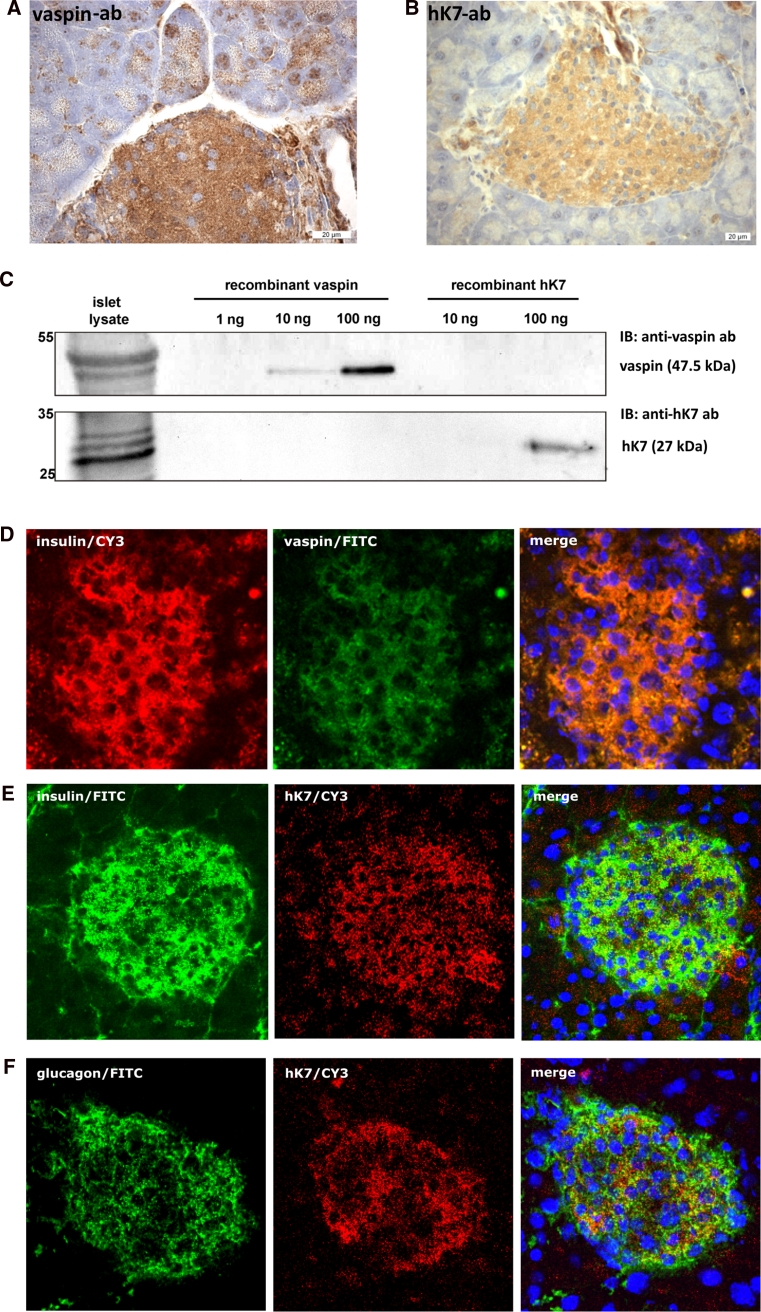
hK7 co-expression with vaspin in pancreatic islets. **a**, **b** Immunostaining of vaspin and hK7 in pancreatic islets of C57BL/6JRj mice. **c** Western blot analysis of vaspin and hK7 protein expression in lysates of isolated islets of female C57BL/6JRj mice with corresponding recombinant control proteins (for male mice, see also Supplementary Fig. S6A). **d** Vaspin and insulin, **e** hK7 and insulin, and **f** hK7 and glucagon staining in pancreatic islets of C57BL/6JRj mice. Double staining of insulin and vaspin by fluorescence immunohistology (merge, *right*) suggests co-localization of both proteins within insulin producing β-cells [**d** insulin (Cy3-channel, *red*), vaspin (FITC-channel, *green*); **e**, **f** insulin (FITC-channel, *green*), hK7 (Cy3-channel, *red*), nuclei (DAPI-channel, *blue*) from *left* to *right*]

mRNA of 8 out of the 15 kallikrein family members is expressed in pancreatic tissue [37]. hK7 expression has previously been reported to be restricted to the (exocrine) pancreas [39, 40]. Immunohistochemical analysis of isolated murine pancreatic islets suggests that vaspin as well as hK7 are expressed in different islet cell types (Fig. 3a, b). Western blot analysis of isolated pancreatic islets from female and male C57BL/6JRj mice corroborate these observations with detectable protein expression of hK7 and vaspin (Fig. 3c; Supplementary Fig. S6A for male littermates) extending previous findings of vaspin mRNA expression in human pancreas [41]. Furthermore, fluorescence immunochemistry indicates that vaspin might co-localize with insulin in pancreatic β-cells (Fig. 3d). Along these lines, we have analyzed pancreatic hK7 localization in parallel with insulin or glucagon (Fig. 3e, f). Co-localization of hK7 with insulin is substantially less clear, and hK7 seems unlikely to co-localize with glucagon in pancreatic α-cells (Fig. 3f). Specificity of the antibodies was confirmed using controls without primary antibodies (Supplementary Fig. S6B, C). Taken together, these data demonstrate co-expression of vaspin and hK7 in pancreatic islets and suggest co-localization of vaspin and insulin in β-cells.

### Vaspin effects on isolated murine pancreatic islets

**Fig. 4:**
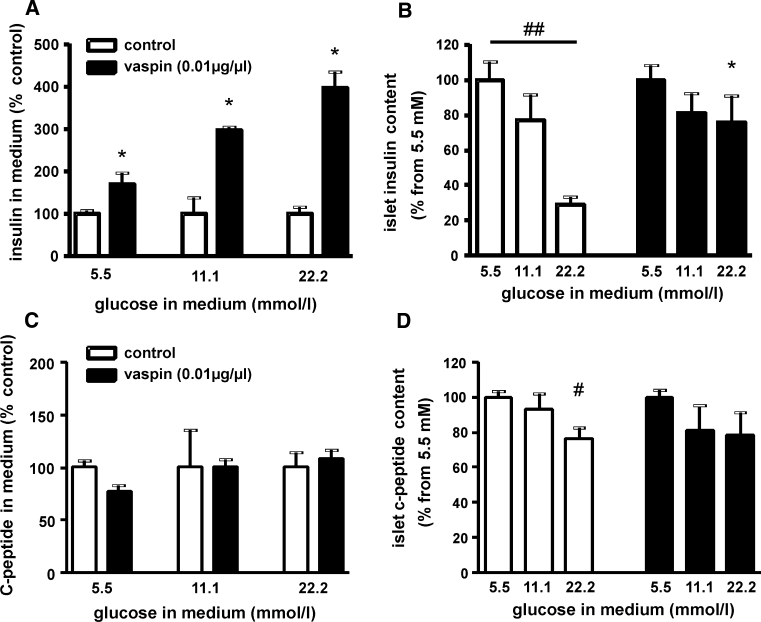
Vaspin effects on isolated murine islets. **a** Islets culture experiments after 2.5 h incubation with increasing glucose concentrations in the medium show significantly higher insulin medium concentrations at all glucose concentration for vaspin (10 μg/ml) treated islets [see also Supplementary Fig. S7A (absolute values) and Fig. S7B (vaspin treatment using 10 ng/ml)]. **b** Insulin content of treated and untreated islets (^#^ *p* < 0.05; ^##^ *p* < 0.01 between different glucose concentrations within treatment groups; *p < 0.05 for vaspin vs. control islets). **c**, **d** Relative C-peptide concentrations in the medium and relative C-peptide islet content at increasing glucose concentrations

Based on these findings, we tested the hypothesis that vaspin protects insulin from hK7-dependent degradation after secretion from the β-cell. Thus, we treated isolated pancreatic islets from C57BL/6JRj mice at different glucose concentrations with recombinant vaspin (10 μg/ml) and measured insulin concentration in the medium and islet insulin content. At a glucose concentration of 5.5 mmol/l, insulin concentrations in the medium were significantly increased in vaspin-treated islets, whereas islet insulin content was not altered compared to control islets (Fig. 4a, b). At higher glucose concentrations (11 and 22 mmol/l), vaspin treatment resulted in an even more pronounced, up to fourfold higher insulin concentration in islet supernatants compared to untreated control islets [Fig. 4a; Supplementary Fig. S7A for absolute values (ng/islet/h)]. Experiments using a lower vaspin concentration (10 ng/ml) gave comparable results (Supplementary Fig. S7B). Meanwhile, C-peptide levels in the supernatants and islet C-peptide content were not different between vaspin-treated and control islets (Fig. 4c, d) indicating that increased insulin accumulation in the medium is due to reduced insulin degradation. Taken together, these observations support the hypothesis that vaspin may protect insulin from local degradation and degradation in the circulation by islet-derived hK7.

### Improved glucose tolerance by vaspin is dependent on its serpin activity and increased insulin levels

**Fig. 5:**
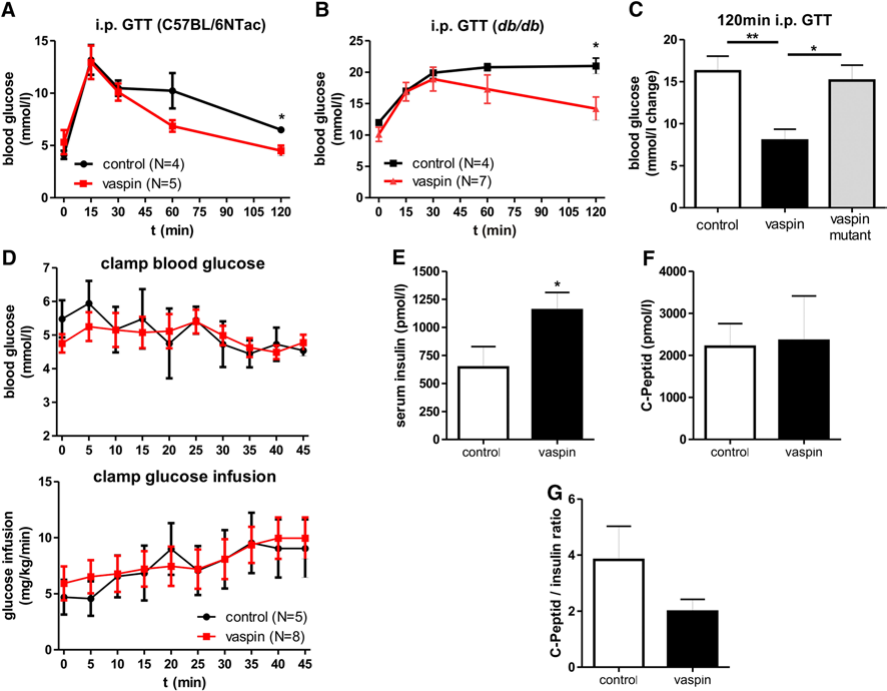
Glucose-lowering effects of vaspin in mouse models depend on serpin activity and are not based on increased insulin sensitivity but increased insulin levels without affecting insulin secretion. **a**, **b** Glucose tolerance during an i.p. GTT is improved after i.p. vaspin treatment (1 mg/kg) in C57BL/6NTac and *db/db* mice. **c** Non-inhibitory vaspinA369P fails to improve glucose tolerance in i.p. GTT after i.p. administration (1 mg/kg) in *db/db* mice. **d** Euglycemic-hyperinsulinemic clamp of female *db/db* mice after vaspin (2 mg/kg) or saline treatment with serum glucose concentrations (*upper panel*) and glucose infusion rate (*lower panel*) during last 45 min clamp. **e**–**g** Vaspin effects on serum insulin and C-peptide levels as well as C-peptide/insulin ratio in *db/db* mice 150 min after a glucose challenge (1 mg/kg vaspin; 2 mg/kg glucose) (*p < 0.05, **p < 0.01)

Recombinant vaspin treatment of diet-induced obese mice has been previously demonstrated to improve glucose tolerance [6]. In addition, we have previously shown that vaspin treatment is associated with sustained glucose-lowering effects in *db/db* mice [8]. Here, we extend these findings by showing improved glucose tolerance after vaspin administration in healthy C57BL/6NTac and in leptin receptor-deficient *db/db* mice (Fig. 5a, b). To investigate, whether serine protease activity is required for vaspin effects on glucose metabolism, we treated *db/db* mice with native and the variant vaspinA369P, which lacks serine protease activity. We find that non-inhibitory vaspinA369P fails to improve glucose tolerance in i.p. GTT in *db/db* mice (Fig. 5c), demonstrating that improved glucose metabolism parameters after vaspin treatment are critically dependent on vaspin serpin activity.

To dissect the suggested role of vaspin as an endogenous insulin sensitizer [6] from its potential role as inhibitor of insulin degradation by hK7, we performed euglycemic-hyperinsulinemic clamp studies in *db/db* mice. Interestingly, glucose infusion rates during the last 45 min of the clamp were indistinguishable between vaspin and saline (control) treated mice (Fig. 5d), suggesting that changes in insulin sensitivity upon vaspin treatment do not represent the mechanism underlying improved glucose tolerance. In subsequent experiments, we found significantly increased insulin serum concentrations (Fig. 5e) in vaspin-treated *db/db* mice compared to saline-treated controls 150 min after a glucose challenge (2 mg/kg body weight). Despite higher post-glucose challenge insulin concentrations in vaspin-treated mice, circulating C-peptide levels were not different in vaspin- and saline-treated mice (Fig. 5f). A tendency for a lower C-peptide to insulin ratio further suggests that insulin is disproportionally higher than C-peptide (Fig. 5g), supporting the hypothesis that vaspin may inhibit insulin degradation in the circulation.

## Discussion

Vaspin was identified as an adipokine with insulin-sensitizing effects, which is predominantly secreted from visceral adipose tissue in a rat model of type 2 diabetes [6] and has been shown to significantly reduce blood glucose concentrations in various mouse models [6, 8]. Therefore, vaspin may represent a novel treatment tool for diabetes intervention strategies.

Here, we provide novel mechanistic and structural insight into previously reported improvement of glucose metabolism after vaspin administration to rodent models of obesity and diabetes [6, 8]. To this point, the serpin family affiliation of vaspin has been based on sequence homology data [6]. We therefore recombinantly expressed human vaspin and determined the X-ray structure after crystallization of the protein confirming the typical structural core domains of serpins consisting of three β-sheets and nine α-helices. Since a broad range of common serine proteases have already been screened for vaspin inhibition [6], we focused on the human kallikrein family previously unrecognized in this context. Circulating plasma levels of tissue kallikrein have been recently shown to be significantly higher in patients with type 2 diabetes compared to individuals with normal glucose metabolism [42]. In addition, hK5 has been reported to be co-localized with vaspin in human skin [25], and a majority of hKs are expressed in the pancreas at least on the mRNA level [37]. Notably, hK7 substrate specificity has been investigated using bovine insulin B chain as substrate [38]. Consequently, we initially screened the closest related members, hK4, hK5 and hK7, for inhibition by vaspin. We found specific and serpin-mechanism-based inhibition of hK7 activity with kinetics of the inhibition reaction supporting a physiological role of the vaspin–hK7 system. We could furthermore isolate vaspin–hK7 complexes from human plasma samples by affinity pull-down after addition of recombinant vaspin. Thus, our results demonstrate that vaspin is a functional serpin inhibiting the serine protease hK7.

hK7 is best known for its role in skin desquamation [43, 44], and there is growing evidence linking hK7 overexpression to endocrine-related malignancies, such as ovarian, breast and testicular cancer [45–47]. Interestingly, the role of hK7 in skin desquamation and recent data on vaspin and psoriasis might point to additional roles for the vaspin–hK7 system in obesity-associated skin disease such as psoriasis [43, 44, 48].

However, the function of hK7 in other tissues or in metabolic diseases is far less understood. Interestingly, the bovine insulin B chain has been used as model substrate in an early study on substrate specificity of recombinant hK7 [38]. Based on these data, we hypothesize that inhibition of hK7 by vaspin may prevent hK7-mediated insulin degradation both in the circulation, as suggested by the detection of vaspin–hK7 complexes in human plasma, and probably already beginning after secretion at the level of pancreatic islets. Formation of a vaspin–hK7 complex could thereby contribute to delayed insulin degradation in the circulation and stabilize circulating insulin concentrations, subsequently leading to improved insulin mediated glucose uptake.

Indeed, we provide in vitro data that human insulin represents a substrate of hK7 with cleavage sites in the A and B chain. Interestingly, hK7 cleavage sites of insulin are the same (Tyr14—A chain and Tyr16—B chain) or immediately next to (Tyr26 vs. Phe24 and Phe25 within B chain) cleavage sites of the major insulin-degrading enzyme (IDE) [49], suggesting that hK7 recognizes the three dimensional structure of insulin away from the insulin receptor binding site and thus might also be able to cleave receptor-bound insulin.

Moreover, vaspin and hK7 are co-expressed in pancreatic islets isolated from C57BL/6JRj mice and vaspin is co-expressed with insulin in murine β-cells, supporting a physiological role of the vaspin–hK7 interaction. Furthermore, these data suggest that the glucose-lowering effects of vaspin might already begin at the level of pancreatic islets. Indeed, we measured significantly increased insulin concentrations in supernatants of isolated pancreatic islets upon vaspin treatment under all glucose concentrations tested. Comparable C-peptide concentrations in the supernatants as well as islet insulin and C-peptide contents between vaspin-treated and control islets very likely exclude an effect of vaspin on glucose-stimulated insulin secretion.

In vivo, vaspin treatment improved glucose tolerance in healthy C57BL/6NTac mice as well as in *db/db* mice. Importantly, GTTs comparing native and non-inhibitory vaspin variants demonstrate that serpin activity is essential for the glucose-lowering effects of vaspin, as the substrate-only variant vaspinA369P failed to improve glucose tolerance during i.p. GTT in *db/db* mice. Vaspin has been previously postulated to improve glucose tolerance by increasing insulin sensitivity [6]. Using hyperinsulinemic-euglycemic clamp studies, we did not find an effect of vaspin administration on insulin sensitivity in *db/db* mice. However, in line with our in vitro findings that vaspin increases insulin medium concentrations secreted from isolated islets, a glucose challenge in vaspin-treated *db/db* mice was associated with significantly increased serum insulin levels 150 min after glucose injection. The increase of serum insulin was not due to enhanced insulin secretion, since C-peptide levels were indistinguishable between vaspin- and saline-treated mice. Moreover, C-peptide to insulin ratio is twofold lower in vaspin-treated mice compared to control littermates. These data further support the hypothesis that vaspin does not increase insulin secretion or sensitivity, but stabilizes insulin plasma concentrations dependent on its serpin activity, most likely by inhibiting the insulin degrading protease hK7.

The study establishing a first valid hK7 ELISA found serum concentrations of human serum hK7 of 2.9 ± 0.9 ng/ml for male and 2.3 ± 0.7 ng/ml for females, with range from 1.5 to 5 ng/ml [50]. Mean serum vaspin concentration have been reported as ~1 ng/ml [10, 11, 51, 52] and serum vaspin ranged from 0.01 to 6.74 ng/ml. Clinical relevance for comparable protease–protease inhibitor serum concentrations has been described in patients with pancreatic cancer, i.e. serum concentrations of the proteases such as trypsin or elastase are in an equimolar range with the corresponding serpins (α_1_-antichymotrypsin, α_1_-antitrypsin) [53]. On the other hand, for the relationship between the serpin PAI-1 and its target protease tPA, ~3- to 5-fold higher serum concentrations have been found for the protease inhibitor compared to the protease [54].

The determined rate constants for the interaction of vaspin and hK7 together with higher hK7 concentrations in the circulation compared to vaspin may limit the physiologic relevance of this system. However, given the range of hK7 and vaspin expression, it is very likely that there exist at least subgroups of the population which have equimolar concentrations of vaspin and hK7. Furthermore, an excess of the protease may represent the healthy, physiologic situation. However, the serpin–protease ratio may change in the course of a disease. For vaspin, we and others have reported that elevated vaspin serum concentrations are associated with obesity and impaired insulin sensitivity in humans [10, 11]. But, so far, influences of obesity or related metabolic diseases on the expression and serum concentration of hK7 are unknown. Furthermore, it has been shown that vaspin expression follows a circadian rhythm, with expression peaks preceding postprandial insulin concentration peaks [55]. In this study, the authors concluded that the reciprocal relationship between serum vaspin and insulin may negate the importance of vaspin as a physiological insulin sensitizer. As we could show, vaspin does not improve insulin sensitivity. But a pre-insulin expression peak of vaspin could acutely change the vaspin–hK7 ratio in favor of a relevant serpin–enzyme interaction and thus positively influence insulin action by providing protection from degradation. We can therefore conclude that, under physiologic conditions, the relevance of the vaspin–hK7 interaction may be limited, but the vaspin–hK7 ratio may change acutely after food ingestion before glucose-induced insulin levels peak or, on the other hand, under chronic conditions of hyperglycemia or other metabolic alterations under which the vaspin–hK7 interaction may become significantly relevant. It is important to note that we can not exclude that the beneficial metabolic effects of vaspin treatment occur via vaspin influencing further or different proteolytic cascades that impact on cell function and insulin levels.

Small compound inhibitors of hK7 would be an adequate tool to address these questions. Recently, three isocoumarin small compound inhibitors for hK7 and hK5 have been reported [56]. However, natural and synthetic isocoumarin compounds have been reported to be rather broad serine protease inhibitors [57]. In line with these findings, the isocoumarin compounds reported by Teixeira et al. [56] are not selective by inhibiting both kallikreins tested, hK5 and hK7, and thus are not suitable. In addition, hK7 itself has several complex cellular effects including regulation of α_5_/β_1_ integrin expression [58].

Noteworthy, the major IDE, a Zn^2+^ requiring metalloproteinase [59] can, due to its nature, be excluded as not being inhibited by vaspin. However, we did not systematically test whether vaspin has direct effects on insulin-degrading enzyme. Since insulin clearance is dependent on binding to its receptor and subsequent internalization, we cannot exclude that vaspin has an effect on insulin binding or internalization. Noteworthy, interference of vaspin with normal insulin binding and degradation could also explain the metabolic effects of vaspin. Therefore, further studies are necessary to test the hypotheses that vaspin has direct effects on IDE and that vaspin may interfere with insulin receptor binding and subsequent internalization.

A major site of insulin degradation is the liver, but also muscle and kidney, with a majority of the insulin degradation occurring intracellularly, dependent on preceding insulin receptor-mediated internalization and clearance from circulation (reviewed in [49]). Taking our findings of hK7 inhibition by vaspin, insulin cleavage by hK7 and serpin activity-dependent vaspin effects on glucose levels in vivo, our data indicate that additional degradation of insulin may be mediated by hK7. hK7-dependent degradation of insulin could be inhibited by vaspin, a process which may occur both at the level of pancreatic islets and in the circulation. Further studies are required to assess the quantitative contribution of insulin degradation in the circulation by hK7 to the net degradation of insulin. In addition, receptors for SECs such as the α_1_-antitrypsin–elastase complex have been reported [60] and shown to activate signal transduction pathways for increased serpin gene expression and to mediate SEC clearance from the circulation [61]. Further investigation is necessaryto identify a possible SEC receptor for the vaspin–hK7 complex and to investigate downstream signaling events potentially supporting already reported vaspin effects or triggering as yet unknown actions.

In summary, our data suggest that a major mechanism for previously reported glucose lowering effects of vaspin is to increase the half-life of insulin rather than improving insulin sensitivity by functioning as an inhibitory serpin for the protease hK7. These findings suggest that the vaspin–hK7 system as a potential novel target for anti-diabetic treatment strategies.

## Electronic supplementary material

Below is the link to the electronic supplementary material.

Supplementary material 1 (PDF 3503 kb)
