# Supplementary material for: Vaspin inhibits kallikrein 7 by serpin mechanism
Source: Cell Mol Life Sci. 2013 Jan 31;70(14):2569–83. doi: 10.1007/s00018-013-1258-8 (PMC3689916; doi:10.1007/s00018-013-1258-8)

Online Resource

Vaspin inhibits kallikrein 7 by serpin mechanism

John T. Heiker[1,4], Nora Klöting[2,4], Peter Kovacs[2], E. Bartholomeus Kuettner[3], Norbert Sträter[3], Stephan Schultz[1], Matthias Kern[2], Michael Stumvoll[2], Matthias Blüher[2*] and Annette G. Beck-Sickinger[1*]

[1] Institute of Biochemistry, Faculty of Biosciences, Pharmacy and Psychology, Universität Leipzig, 04103 Leipzig, Germany
[2] Department of Internal Medicine, Universität Leipzig, 04103 Leipzig, Germany
[3] Center for Biotechnology and Biomedicine, Institute of Bioanalytical Chemistry, Faculty of Chemistry and Mineralogy, Universität Leipzig, 04103 Leipzig, Germany

Supplementary Figure S1

Analytics of purified recombinant vaspin, photograph of analyzed vaspin crystals, superposition of Cα-traces of vaspin and α1- antitrypsin and sequence alignment of vaspin and classical serpins. (A) SDS-Page analysis of purified recombinant vaspin (lane 1 whole cell lysate, lane 2 purified vaspin). (B) Western blot of purified vaspin using anti-vaspin antibody. (C) Analytical HPLC of purified vaspin (water/acetonitrile gradient with 0.1% TFA from 20-80% in 30 min. (D). MALDI-TOF spectrum of vaspin in linear mode ($[M_{avg}+H]^{1+}_{theoretical}$=47648. Together with the singly charged ion doubly, triply and quadruply charged ions are observed ($[M_{avg}+2H]^{2+}_{theor.}$=23825, $[M_{avg}+3H]^{3+}_{theor.}$=15883, $[M_{avg}+4H]^{4+}_{theor.}$=11912). (E) Vaspin crystals of space group C2 as used for structure determination. (F) Superposition of alpha-carbon traces of vaspin (red – α-helical, blue – β-fold, grey – loop region) and α1- antitrypsin (black, PDB id 1QLP [1]) as the best matching known protein structure (DALI [2], rmsd = 1.7 Å for 362 out of 375 residues, 41 % sequence identity). Vaspin residues shown in orange are flexible in the crystal structure. (G) Sequence alignment of vaspin, antitrypsin, antithrombin and antichymotrypsin correlated with structural features of vaspin (UniProt numbers: vaspin - Q8IW75, antitrypsin - P01009, antithrombin - P01008, antichymotrypsin - P01011). Sequences are without signal peptide. Letter/color code: white letters/black background - fully conserved, white letters/grey background - mostly conserved, black letters/grey background - often conserved.

Supplementary Figure S2

hK7 inhibition by vaspin: stoichiometry of inhibition and association rate constant, k_{ass}. (A) The stoichiometry of inhibition of factor hK7 is taken as the x-intercept of the linear regression when residual protease activity is plotted against the ratio of vaspin to protease. (B) Determination of association rate constant, k_{ass}, of hK7 inhibition by vaspin. k_{ass} was determined by plotting ln[remaining activity] vs. time of incubation. The constant k_{ass} was calculated based on the equations $\ln [E] = -k_{obs} \cdot t$ and $k_{ass} = k_{obs}/[I]$. Data shown are representative of at least four independent experiments.

Supplementary Figure S3

Variant vaspinT365R is unable to form serpin-hK7 complexes and serves as substrate only as demonstrated by SDS-PAGE and Western Blot analysis. Data shown are representative of at least two independent experiments.

Supplementary Figure S4

MALDI-TOF analysis of vaspin-hK7 complex formation and P1 residue determination. (A) Predicted cleavage site of hK7 in vaspin RCL sequence and resulting C-terminal fragment vaspin(379-414). (B) Linear mode MALDI-TOF spectra from vaspin-hK7 incubation mixture (top) and vaspin control (bottom). Peaks are corresponding to hK7 band (27 kDa), cleaved vaspin (43.6 kDa), native vaspin (47.6 kDa) and the vaspin-hK7 complex (70 kDa). Doubly charged ion peaks of vaspin (23.8 kDa) and cleaved vaspin (21.8 kDa) are also observed. (C) MALDI-TOF spectrum of the C-terminal vaspin fragment in reflector mode ($[\text{vaspin(379-414)+H}]^{1+}_{\text{theor.}}$=4037.651) and detailed isotope pattern of the ion peak used for MSMS analysis.

Supplementary Figure S5

MALDI-TOF analysis of insulin degradation by hK7. (A) Amino acid sequence of insulin A and B chain with intra and inter chain disulfide bonds and identified cleavage sites. Identified fragments are indicated according to MS peaks in (B). (B) MALDI-TOF analysis of control insulin and hK7 digested native insulin after 60 min and 24 h. Primary cleavage sites after 60 min corresponding to cleavage after tyrosine 16 in insulin B chain and tyrosine 14 in insulin A chain ([insulin B chain $(F^{1}\text{-}Y^{16})+H]^{1+}_{\text{theoretical}}$=1617.84 (1), [insulin B chain $(L^{17}\text{-}T^{30})+H]^{1+}_{\text{theoretical}}$=1829.92 (2), [insulin A chain $(G^{1}\text{-}Y^{14})+Na]^{1+}_{\text{theoretical}}$=1540.64 (3)). A secondary cleavage site after tyrosine 26 of insulin B-chain is observed after prolonged incubation for 24 h ([insulin B chain $(L^{17}\text{-}Y^{26})+H]^{1+}_{\text{theoretical}}$=1190.59 (4)). Peak identities were confirmed by MALDI-TOF-MSMS analysis of insulin fragments.

Supplementary Figure S6

Western blot analysis of isolated islets and negative controls for fluorescence immunohistology of murine pancreas slides. (A) Vaspin protein expression in the lysate of isolated islets of male C57BL/6JRj mice detected by anti vaspin antibody with recombinant vaspin as control. (B) Insulin and (C) glucagon double staining with secondary antibody controls in pancreatic islets of C57BL/6JRj mice by fluorescence immunohistology (B: insulin (Cy3-channel, red) and secondary antibody control (FITC-channel, green); C: glucagon (FITC-channel, green) and secondary antibody control (Cy3-channel, red); B+C: nuclei: DAPI-channel, blue).

Supplementary Figure S7

Vaspin treatment of isolated pancreatic islets form C57BL/6JRj mice (A) Absolute values for insulin release (ng/islet/h) and insulin islet content (ng/islet) corresponding to Figure 6A and B. (B) Absolute values for insulin release (ng/islet/h) and islet content (ng/islet) for vaspin treated islets (10ng/ml). *, $p < 0.05$ for vaspin versus control.

Supplementary References

1. Dementiev A, Simonovic M, Volz K, Gettins PG (2003) Canonical inhibitor-like interactions explain reactivity of alpha1-proteinase inhibitor Pittsburgh and antithrombin with proteinases. J Biol Chem 278 (39):37881-37887

2. Holm L, Kaariainen S, Rosenstrom P, Schenkel A (2008) Searching protein structure databases with DaliLite v.3. Bioinformatics 24 (23):2780-2781

Supplementary Figure 1

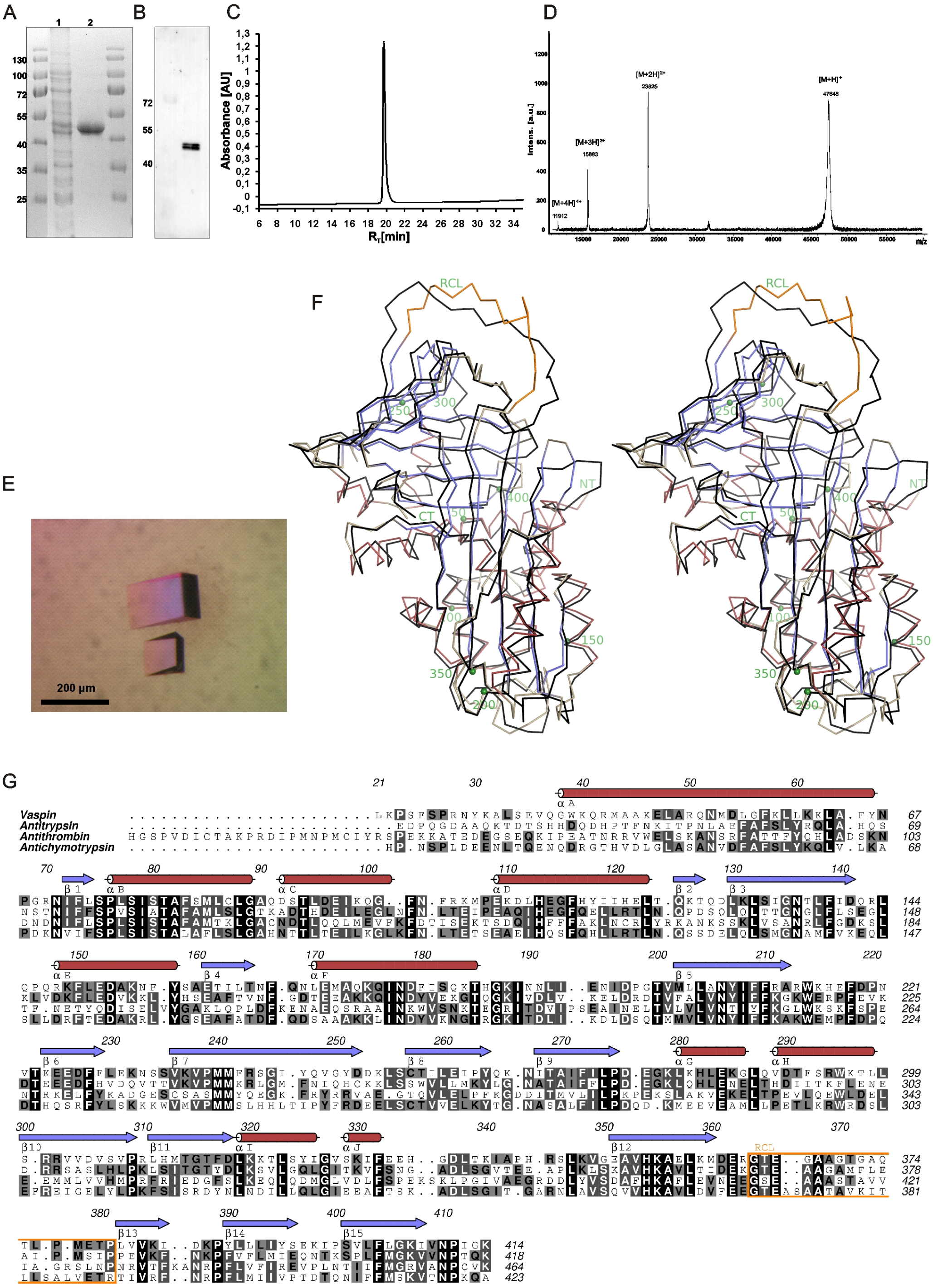

Supplementary Figure 2

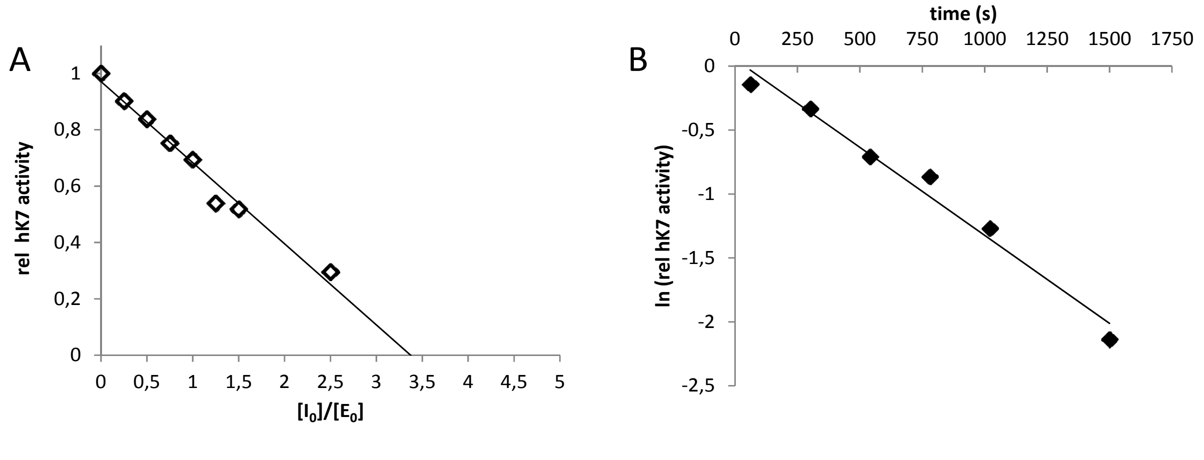

Supplementary Figure 3

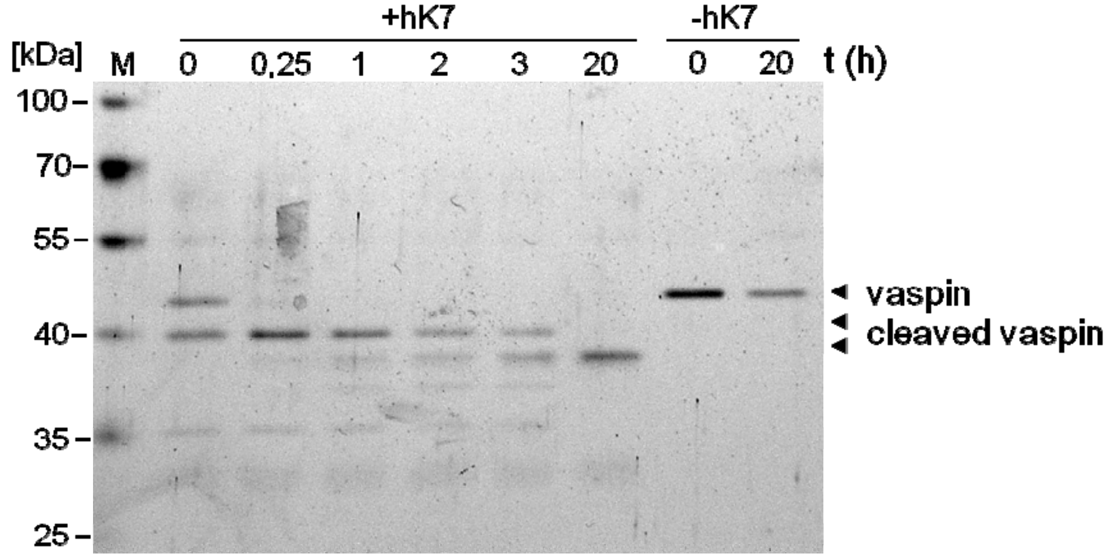

Supplementary Figure 4

A **G**[364]TEGAAGTGAQTLP**M**[378]- -**E**[379]TPLVVKIDKPYLLLIYSEKIPSVLFLGKIVNPIG**K**[414] [Vaspin(379-414) + H]$^{1+}_{theor.}$ = 4037,651

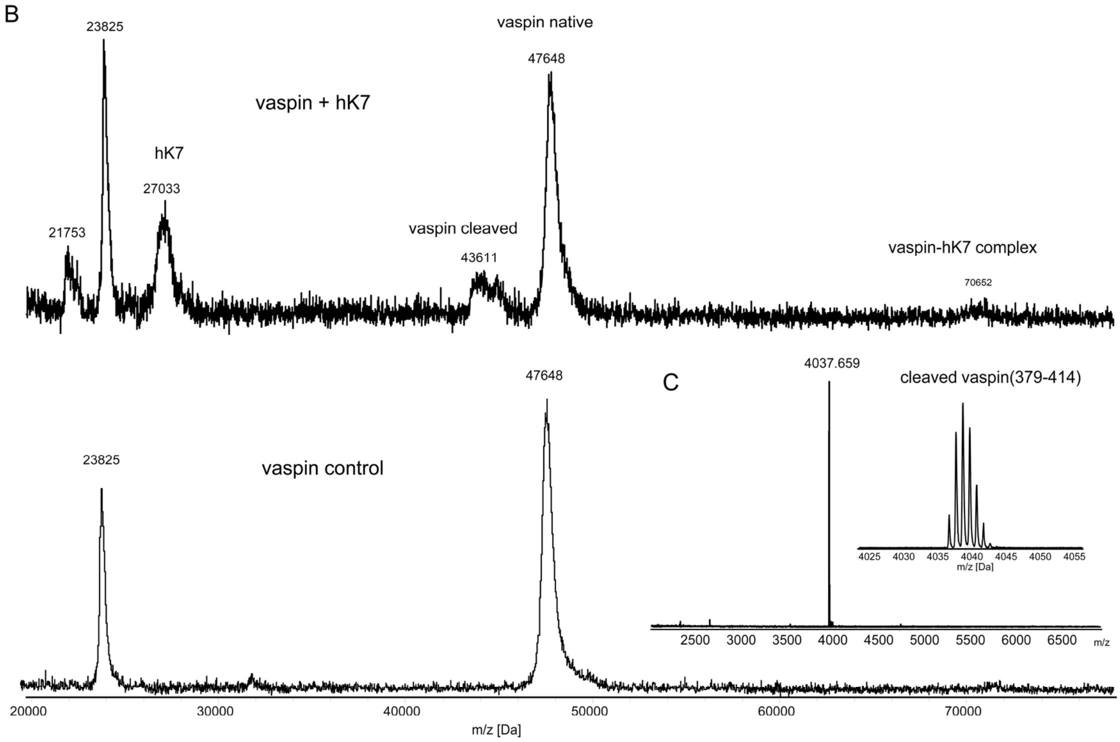

Supplementary Figure 5

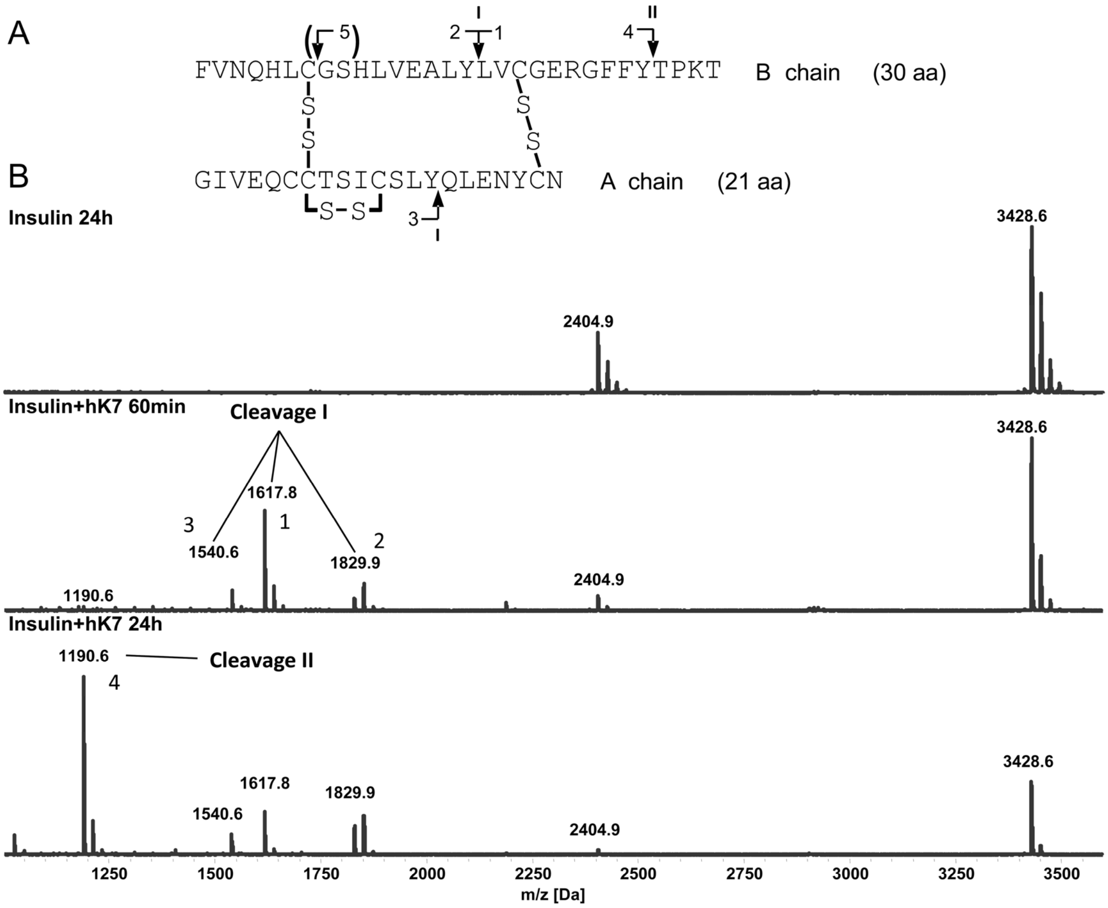

Supplementary Figure 6

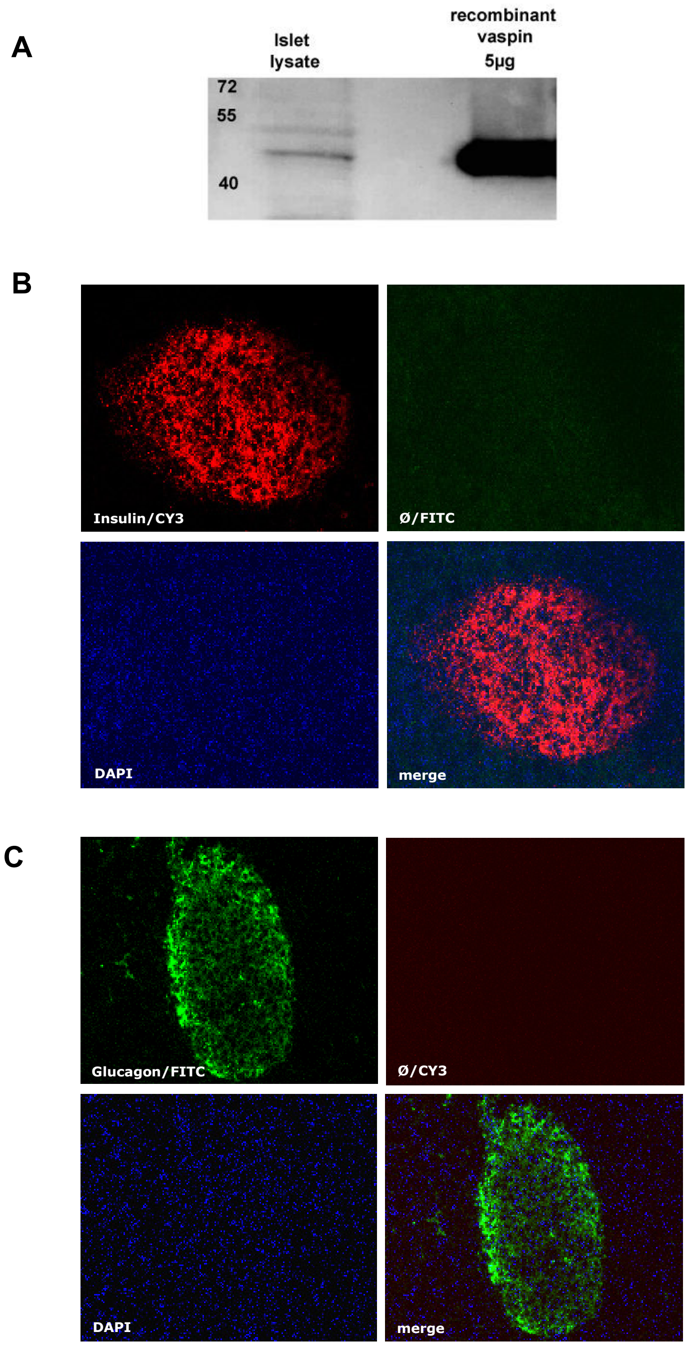

Supplementary Figure 7

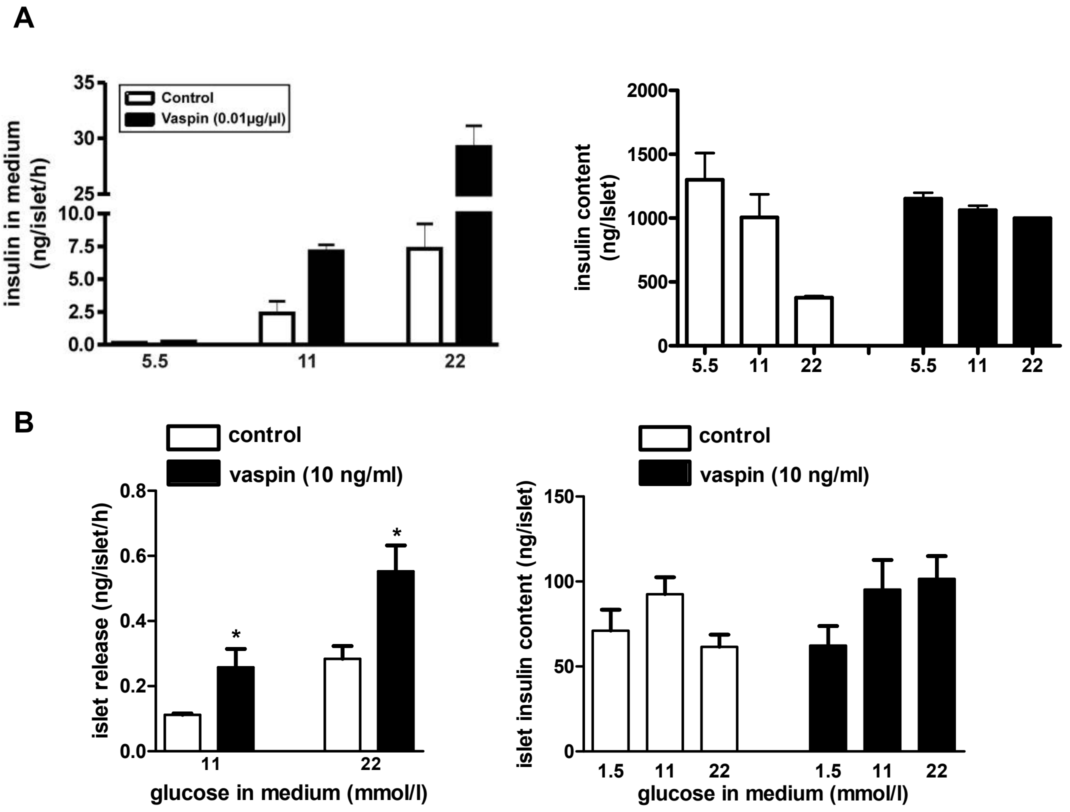

Supplement: Supplementary file 1 — Supplementary material 1 (PDF 3503 kb) [file 18_2013_1258_MOESM1_ESM.pdf]
